# Physical and Chemical Barriers in the Larval Midgut Confer Developmental Resistance to Virus Infection in *Drosophila*

**DOI:** 10.3390/v13050894

**Published:** 2021-05-12

**Authors:** Simon Villegas-Ospina, David J. Merritt, Karyn N. Johnson

**Affiliations:** Faculty of Science, School of Biological Sciences, The University of Queensland, Brisbane 4072, Australia; s.villegasospina@uq.edu.au (S.V.-O.); d.merritt@uq.edu.au (D.J.M.)

**Keywords:** larval development, antiviral mechanisms, *Drosophila*, dicistrovirus, midgut, peritrophic matrix, gut pH

## Abstract

Insects can become lethally infected by the oral intake of a number of insect-specific viruses. Virus infection commonly occurs in larvae, given their active feeding behaviour; however, older larvae often become resistant to oral viral infections. To investigate mechanisms that contribute to resistance throughout the larval development, we orally challenged *Drosophila* larvae at different stages of their development with Drosophila C virus (DCV, *Dicistroviridae*). Here, we showed that DCV-induced mortality is highest when infection initiates early in larval development and decreases the later in development the infection occurs. We then evaluated the peritrophic matrix as an antiviral barrier within the gut using a Crystallin-deficient fly line (*Crys*^−/−^), whose PM is weakened and becomes more permeable to DCV-sized particles as the larva ages. This phenotype correlated with increasing mortality the later in development oral challenge occurred. Lastly, we tested in vitro the infectivity of DCV after incubation at pH conditions that may occur in the midgut. DCV virions were stable in a pH range between 3.0 and 10.5, but their infectivity decreased at least 100-fold below (1.0) and above (12.0) this range. We did not observe such acidic conditions in recently hatched larvae. We hypothesise that, in *Drosophila* larvae, the PM is essential for containing ingested virions separated from the gut epithelium, while highly acidic conditions inactivate the majority of the virions as they transit.

## 1. Introduction

Infections caused by insect-specific viruses have a wide range of outcomes, from high lethality to asymptomatic infections with little or no impact on the insect host [1,2]. The different outcomes depend in part on the life stage at which infection occurs. Virus infections in larvae tend to cause lethality more often than in adults [3,4,5,6,7]. Moreover, within the different larval stages there can be variation in the resistance or susceptibility to the viral infection [3,8]. While the use of insect-specific viruses as biocontrol agents provides a target-specific alternative to chemical-based methods and has indeed become common practise in the agricultural field [9,10,11,12], the stage-specific variability in the outcome of infection can be a major challenge [3,4,6,12]. For example, baculoviruses often cause lethal infections when orally delivered to larvae of Lepidopteran pests, but in many cases, significant mortality is achieved only when the virus is fed to newly hatched or early-instar larvae [13]. The interactions and mechanisms behind the increasing resistance to viruses throughout larval development are complex, based not only on the increasing size and feeding behaviour of the host [5,7,14], but on the balance between physical, chemical, and physiological factors [15,16,17,18], many of which are still being elucidated.

In nature, a large proportion of viruses initiate infection via ingestion. The gut represents a large surface contact area and has substantial defence mechanisms [16,18,19,20]. How these defence mechanisms function against orally acquired viruses has been studied in a number of different virus–host pairs, revealing both general, and species- or virus-specific responses. In insects, the peritrophic matrix (PM) is a complex 3D structure of chitin, glycoproteins, and proteoglycans that serves as a first physical barrier to foreign ingested contents [21,22,23,24], serving a similar function as the mucosal layers secreted by the mammalian epithelia [25]. Across several insect families, the PM is constitutively secreted by specialised cells at the opening of the midgut (the cardia) to engulf and isolate the food bolus as it enters, preventing any direct contact with the epithelial cells, while still allowing for the exchange of enzymes and digested contents [21,26]. The compartmentalisation of the midgut generates gradients in pH, ions, and digestive enzymes [27,28,29,30]. Throughout its length, the complexity and permeability of the PM can vary, allowing for the exchange of different products of the digestion into the gut lumen [31,32,33,34]. Furthermore, the interplay between the permeability of the PM, the chemical conditions in the gut, and the presence of resident microorganisms and their products is essential for maintaining gut homeostasis in terms of growth and repair signals, and effective cellular and humoral defence responses [2,18,35]. Little is known however about how the structure and composition of the PM varies throughout the life stages, and how effective its contribution may be regarding the containment of viral particles.

Early studies of the *Drosophila*-specific viruses indicate that larvae of *D. melanogaster* become more resistant to Drosophila C virus, or DCV, (*Picornavirales*, *Dicistroviridae*, *Cripavirus* [36,37,38]) as they age [39,40]. Indeed, rearing the larvae in media that was contaminated with DCV at different stages of their development would influence how many survived to become adults: the older they were challenged, the more would survive [40]. The *Drosophila*–DCV model serves as a valuable model to explore aspects of insect development and antiviral responses to natural routes of viral infection [41,42], and to understand general mechanisms by which viruses may overcome the defences of the gut. Furthermore, the biology of RNA viruses is a field of increasing interest given their own potential for biocontrol of insect pests [43,44,45], but also by their importance in the decline of economically important insects [46,47].

## 2. Materials and Methods

### 2.1. Virus, Fly Lines, and Husbandry

The Ellis Beach (EB) isolate of DCV was used in this study [37]. For tissue culture assays, we used aliquots of purified DCV in 0.1 M phosphate buffer (PB), pH 7.2 [37], kept at a concentration of 2.5 × 10^9^ IU/mL (10^9.4^ IU/mL). For oral feeding assays, we used lysates of adult *w*^1118^ flies that had been previously infected by microinjection (Nanoject II, Drummond Scientific, Broomall, PA, USA) with 50 nL of our DCV stock, diluted to 10^8^ IU/mL in sterile PBS, or mock infected with sterile PBS, and collected and frozen at 3 days post-infection (dpi). For every time point and biological replicate of each larval or adult feeding assays, we prepared appropriate volumes of fresh lysates in a Tissuelyser II (Qiagen, Hilden, Germany), maintaining a proportion of 15 µL of sterile PBS per fly, followed by two 5 min centrifugations at 14,000× *g* to clear any debris and bacterial contamination. Final viral titres ranged between 10^9^ and 10^10^ IU/mL (*N* = 10 lysates, randomly selected across all experiments; data not shown [48]).

The *Crys*^−/−^ line (*w*^1118^; Mi{ET1}Crys^MB08319^) was obtained from the Bloomington Drosophila Stock Center (BDSC-26106) at Indiana University Bloomington for this study. The *w*^1118^ (BDSC-5905) and Champetières (Kyoto-103403) lines have been maintained for several years in our research group. All fly lines were cleared of potential viral infections by dechorionation of embryos, and tested for *Wolbachia* infection, as previously reported [49]. When necessary, *Wolbachia* infection was cleared by rearing a generation under 0.03% *w*/*v* tetracycline treatment [50], and clearance was confirmed after at least 4 generations by standard PCR as reported elsewhere [49]. Our fly stocks were kept in standard cornmeal media, in virus-free incubators at 25 °C with a 12 h light–dark cycle.

### 2.2. Larval Oral Infection

To test the susceptibility of *Drosophila* larvae to DCV oral infection, young adult flies were left to lay eggs for 12–18 h on a thick yeast paste placed on 2.5% *w/v* apple juice agar plates. Eggs were collected, cleaned in 1:50 diluted bleach (0.25% *v/v* sodium hypochlorite) for 1 min, thoroughly washed with distilled water and transferred to 10 cm rearing Petri dishes with cornmeal *Drosophila* food. Four time points were chosen relative to the time the majority of eggs started hatching: L0 (egg-sized larvae, 0–6 h post-hatching), L1 (12 h after L1, clearly defined mouthpieces), L2 (24 h after L1), and L3 (24 h after L2). Based on the larval development at 25 °C and on the observed physiological structures of the mouthpieces and spiracles [51], our L1, L2, and L3 time points corresponded to each of the three defined larval instars. Furthermore, L0 was chosen as natural DCV infection is believed to initiate during, or soon after, the larva hatches. At each time point, we transferred an excess of larvae from the rearing plates into size-appropriate droplets of a 4:1 mix of DCV- or PBS-injected fly lysates with a yeast–PBS solution, stained with blue food colouring. These droplets were placed on a clean, sterile 3.5 cm Petri dish, and the larvae were left to feed ad libitum for 3 h. After this period, we collected groups of at least 25 larvae, whose guts were stained in blue as a confirmation of having ingested the lysate, into fresh 3.5 cm Petri dishes with Drosophila food, and let them pupate and emerge as adults. Each biological assay was repeated at least 4 independent times, and included 2 groups of PBS-fed and 3 groups of DCV-fed larvae.

### 2.3. Gut Volume Measurements

To estimate the size of the gut based on an ingested amount, we obtained L1, L2, and L3 larvae simultaneously, by passing adult flies into fresh 3.5 cm cornmeal Petri dishes every day for 3 days. We transferred groups of larvae of varying sizes onto a droplet of a solution of 40 kDa FITC-labelled dextran (Sigma-Aldrich, St. Louis, MO, USA), diluted to 1 mg/mL with a yeast–PBS solution, spread on a clean Petri dish. Larvae were left to feed for at least an hour in the dark, which is sufficient time for the whole gut to be filled with the FITC-labelled particles. Groups of 5 similarly sized larvae of the same instar (based on morphology) were collected, and their weight was measured on an analytical scale (0.1 mg error). Any excess FITC on the surface was washed with cold PBS, the pool of larvae was crushed with a pestle in 120 µL of sterile PBS, and pulse-pelleted to remove debris. We measured the fluorescence signal of FITC in a Typhoon FLA9500 (GE Healthcare Life Sciences, Milwawkee, WI, USA) from two 50-µL aliquots per sample, which we added into separate wells of a 96-well polypropylene plate (Sarstedt, Nümbrecht, Germany) containing 50 µL PBS per well for a 1:1 dilution, and compared the obtained values to standard curves of the FITC–yeast mix used per assay. We used samples of each instar fed with yeast–PBS only, and equivalent yeast–PBS standard curves to correct for the background auto fluorescence of the yeast and the larval tissues.

### 2.4. Adult Oral Infection

To evaluate whether a compromised PM would impact the susceptibility of adult flies to oral DCV infection, we collected 3–7 day old males and females of *w*^1118^ and *Crys*^−/−^ in pools of 18–20. Flies were starved for 6 h, and left to feed for a day on a 1 cm^2^ piece of filter paper soaked with 50 µL of a 4:1 solution of DCV- or PBS-injected lysates with 10% sucrose in PBS (final sucrose concentration = 2%) stained with blue food colouring. After this, they were transferred to fresh cornmeal vials. Four adults per treatment were collected for RNA extractions before and after starvation, to rule out any effect starvation could have over the transcription of *Crys* mRNA, and after 6 h of DCV or PBS feeding. Mortality was scored daily over a 30-day period, and deaths within the first 24 h after the oral challenge were assumed to be due to starvation and stress. We conducted 3 biological replicates of this experiment, each containing 1 PBS-fed and 2 DCV-fed replicates per sex, per line.

### 2.5. PCR and RT-qPCR

To screen for *Wolbachia* infection and to validate the Mi{ET1} insertion in the *Crys* gene for the *Crys*^−/−^ line (Figure 3A), we obtained genomic DNA from pools of 4 flies using a modified protocol of the Viral RNA Extraction kit (Qiagen, Hilden, Germany) [52]. We used the ThermoPol^®^ kit (New England Biolabs, Ipswich, MA, USA) per the manufacturer’s instructions with 5 mM MgCl_2_ for conventional PCR amplification: 95 °C, 3 min; 35 × 95 °C 30 s-52 °C (*12S*, *Wsp*) or 57 °C (*Crys*), 30 s-72 °C 1 min; 72 °C; and 5 min final extension, using the primers reported in Table 1. Products were verified in 1.5% agarose gels stained with SYBR^TM^ Safe (Thermo Fisher Scientific, Waltham, MA, USA).

To quantify the mRNA expression of genes, we first obtained RNA from pools of adults or larvae using TRIzol^®^ (Invitrogen, Carlsbad, CA, USA) reactions, following the manufacturer’s instructions. We DNAse-treated the RNA (RQ1 DNAse, Promega, Madison, WI, USA), and synthesised cDNA using a mix containing 1 pg of each specific reverse primer per 1 µg RNA, and using the SuperScript^TM^ III first strand cDNA kit (Thermo Fisher Scientific, Waltham, MA, USA), following the manufacturer’s instructions. We then amplified the cDNA samples by standard qPCR cycling (94 °C, 2 min; 40 × 94 °C, 10s-57 °C, 20s-72 °C, 30s; Melt 65–95 °C), using duplicate reactions of Platinum SYBR Green qPCR SuperMix-UDG (Thermo Fisher Scientific, Waltham, MA, USA) following the manufacturer’s instructions and the primers reported in Table 1. The relative expression was calculated by the ΔΔCt method, using the specific amplification efficiency of each pair of primers. The efficiency had been previously calculated by the amplification of serial dilutions of a positive sample.

### 2.6. Microscopy

To measure the permeability of the gut to DCV, we fed L1 and L3 larvae of *w*^1118^ or *Crys*^−/−^ flies with 500 kDa FITC-labelled dextran beads (Sigma-Aldrich, St. Louis, MO, USA). These beads are approximately the same size as the 30 nm DCV virions (α = 0.33*MM0.46, where α is the radius (Å) of a spherical dextran particle, and MM is its molecular weight (g/mol) [53,54]; 2α ≈ 28 nm for 500 kDa dextran beads). We then dissected whole guts in 1:1 glycerol-PBS over ice-cold microscope slides, and fresh mounted for immediate microscopy. Three regions of the midgut (anterior, middle, and posterior) were quantified for FITC signals in the intra-PM space and into the gut lumen. In samples where the gut epithelium ruptured during dissection, thus expelling part of the PM, the neighbouring area was excluded. Furthermore, the hindgut was excluded from analyses as (a) it is not clear whether the PM is always present in this region; and (b) the Pax6-EGFP present in the Mi{ET1} insertion as a brain and eye selection marker for the *Crys*^−/−^ flies is also transcribed in the hindgut and anal plates of larvae (Figure 3B) and adults [48]), interfering with the FITC signal.

For the confirmation of the pH gradients of the gut, we fed L0/L1 or L2/L3 larvae of *w*^1118^, Champetières, and *Crys*^−/−^ (*N* = 7 per line, per group), with a 1:1 mixture of yeast-in-PBS and 0.2% *w/v* aqueous *m*-cresolsulphonphtalein (Sigma-Aldrich, St. Louis, MO, USA) for 1–2 h. The larvae were dissected in room temperature 1:1 glycerol-PBS, and immediately observed under bright field microscopy. As previously reported, once dissected, the extreme pH values of the gut are rapidly neutralised (less than 3 min) [27]. Imaging of the orange-red acid region and the yellow-purple neutral-alkaline gradient in Figure 5A appears less acidic or alkaline than immediately after dissection and is used for illustrative purposes only. Slides were visualised in a Nikon Eclipse 50i (Minato, Tokyo, Japan) fluorescence microscope. Images were taken with a Nikon 3DSL camera under bright field, or GFP/FITC epifluorescence filters. We used Adobe Photoshop CS6 (Adobe Inc., San Jose, CA, USA) to merge the obtained images when needed, and for adjustment of saturation and background.

For transmission electron microscopy (TEM) of middle and posterior midguts of L3 *w*^1118^ and *Crys*^−/−^ larvae, samples were dissected in 1:1 glycerol-PBS over cold slides, immediately fixed in 2.5% glutaraldehyde in PBS, and then in 1% osmium tetroxide, before being dehydrated in a series of ethanols (30%, 50%, 70%, 90% and 2 × 100%). They were infiltrated with Epon^TM^ resin (75% ethanol: 25% Epon; 50% ethanol: 50% epon; 25% ethanol: 75% epon; 2 × 100% Epon) and polymerised at 60 °C for 48 h. All steps except the initial fixation were performed in a Pelco Biowave (Ted Pella, Inc., Redding, CA, USA) according to manufacturers’ instructions. Samples were cut with a UC6 ultramicrotome (Leica, Wetzlar, Germany) at 60–70nm, placed on copper grids and contrasted with uranyl acetate and lead. The sections were imaged in a Hitachi HT7700 (Chiyoda, Tokyo, Japan) transmission electron microscope operated at 80 kV.

### 2.7. Cell Culture

To test the stability of DCV under extreme pH conditions, we mixed independent 5 µL aliquots of DCV stock kept in 0.1 M PB pH 7.2 (2.5 × 10^9^ = 10^9.4^ IU/mL), with 45 µL of either 0.1 M sodium citrate acid buffers (pH 1.0, 3.0, 4.5, or 7.0), 0.1 M sodium bicarbonate alkaline buffers (pH 7.5, 8.5, 10.5, 12), or a PB control, and incubated them for 15 min at room temperature. We then made 10-fold serial dilutions of each treatment with complete cell media (Schneider’s Drosophila media with 10% FBS, 1X Penicillin/Streptomycin, 1X L-glutamine; Gibco, Thermo Fisher Scientific, Waltham, MA, USA) and inoculated Drosophila S2 cells previously seeded into 96-well cell culture plates. The tissue culture infectious dose 50% (TCID_50_) was calculated by the Reed–Muench method [55]. Given the biological variation seen in TCID_50_ assays, each pH treatment was replicated 5 times, each one with its respective uninfected and buffer sterility controls. Furthermore, we measured with a pH-meter the buffering capacity of the solutions used, by incubating 9 volumes of each citrate or bicarbonate solution with 1 volume of PB for 20 min; for each case, the pH would shift no more than 0.2 towards 7.2. 

### 2.8. Statistical Analysis 

For each larval oral infection assay, we calculated the larva-to-adult mortality as the proportion of the number of emerged adults from the number of larvae collected per treatment plate (as percentage values), at each of the four time points. We then used a two-way ANOVA in GraphPad Prism 9.0 (GraphPad Software, San Diego, CA, USA) to calculate the effect and interaction of the oral challenge treatment and developmental time at which the larvae were challenged, over the larva-to-adult mortality. Šídák’s test was used for multiple comparisons of means within groups (PBS vs. DCV, at each time point) to determine which instars are susceptible to lethal DCV infection. Tukey’s test was used for comparing the individual treatment means between groups (time point vs. time point, at either PBS or DCV) to evaluate the difference of DCV mortality when infection occurs earlier or later in development.

To analyse the survival data of adult oral infection assays we used the coxme package [56,57] in R 4.0.3 to fit a Cox mixed-effects model. Results for males and females were analysed separately, each with the fixed effects of mutation and infection, and the random effects of biological and technical replicates. 

Using the fluorescence values obtained from the FITC-feeding assay, we calculated the average volume of the gut in each group of 5 larvae, based on the dilution factor between the larvae lysate measured and the standard concentration (1 mg/mL) of the FITC–dextran–yeast mix used to feed them. We further calculated a relative ratio between gut volume and body mass to estimate whether the gut maintains a similar proportion within the body of the developing larva. We used a Pearson test in GraphPad Prism 9.0 to evaluate the correlation between volume and body mass, and between ratio and body mass.

For RT-qPCR data the expression of target genes relative to the reference gene RpL32 was estimated by comparative quantitation using the amplification efficiency for each qPCR primer set. We used a two-way ANOVA in GraphPad Prism 9.0 to calculate the effects of sex and starvation or infection over *Crys* mRNA expression in adults.

For each of the three midgut regions (anterior, middle, and posterior), the proportion of events where the DCV-sized FITC-dextran permeated into the lumen were compared using χ^2^ tests in GraphPad Prism 9.0 in two ways: between the two instars of a same line, or between the two lines at a given instar.

The DCV titre after incubation under acidic or alkaline conditions was estimated using the Reed–Muench method, and log-transformed for analysis using a one-way ANOVA. Šídák’s multiple comparisons test was then used to evaluate each treatment against the PB-treated control.

## 3. Results

### 3.1. Mortality Decreases When Oral DCV Infection Occurs Later in Larval Development

**Figure 1:**
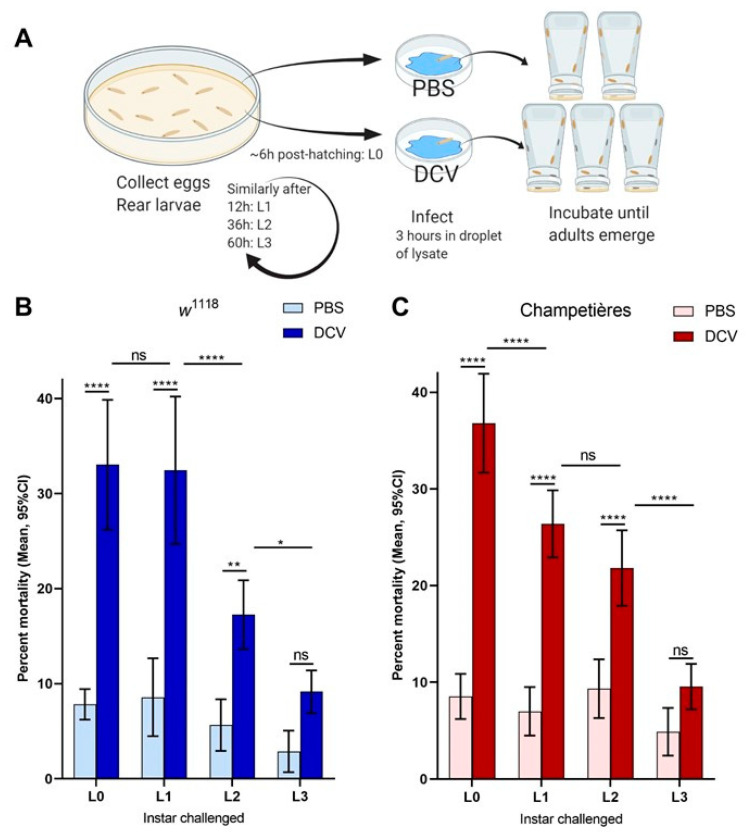
Mortality caused by DCV decreases when infection occurs later in larval development: larvae of the *w*^1118^ and Champetières fly lines were picked from rearing plates and orally challenged with lysates of DCV- or PBS-injected flies at 4 different times post ecclosion, and transferred to individual test plates (**A**). At least 4 biological replicates were done, each containing 2-PBS- and 3-DCV-challenged groups of larvae, per time point. Mortality was calculated from the proportion of adults that emerged from the larvae transferred to each test plate, for each PBS or DCV feeding challenge. A two-way ANOVA analysis was used to compare the effect of infection and the time (of larval development) at which the challenge was done. Using Šídák’s test for multiple comparisons, the survival between PBS and DCV at each time point was compared, to determine that L0, L2, and L2 larvae were susceptible to lethal DCV infection compared to their uninfected controls; and Tukey’s test was used to compare the mortality after PBS or DCV feeding across instars, to determine that the mortality caused by DCV decreased significantly between the L0-L1, L1-L2, and/or L2-L3 times of infection ((**B**,**C**); statistical analysis results are detailed in Table S1; * *p* < 0.05, ** *p* < 0.01, *** *p* < 0.001, **** *p* < 0.0001).

To evaluate the effect of oral DCV infection on the survival of *Drosophila* larvae we set up an oral DCV infection protocol for larvae (Figure 1A). Larvae were challenged with DCV at different developmental times and survival to adult occlusion was scored. Two wild-type fly lines, *w*^1118^ and Champetières, were used for this experiment. The larvae were fed a solution of yeast supplemented with lysates of PBS- or DCV-injected flies. Feeding challenges were carried out in groups of larvae, aged to one of four different times during larval development: 0–6 h old (L0), and 12 (L1), 36 (L2), and 60 (L3) h after this time. In both fly lines, the oral DCV challenge increased the mortality when compared to their respective PBS-fed controls, when larvae were fed with DCV at the L0, L1, and L2 time points (Šídák’s multiple comparisons for PBS–DCV; *p* < 0.0001 for L0 and L1 in either line; *p* = 0.0029 in *w*^1118^ L2, *p* < 0.0001 in Champetières L2), but not at L3 (*p* = 0.2200 for *w*^1118^, *p* = 0.2008 for Champetières) (Figure 1B,C, Table S1). Interestingly, the mean difference in mortality between DCV- and PBS-fed larvae decreased the later in development the DCV challenge occurred (L0 > L1 > L2 > L3; 25% > 24% > 12% > 6% in *w*^1118^; 28% > 20% > 12% > 5% L3 in Champetières; Table S1), indicating that an increase in resistance to oral DCV infection occurs during the development of *Drosophila* larvae, consistent with earlier studies [40].

### 3.2. Body Size Does Not Explain the Increased Resistance Phenotype in Drosophila Larvae

**Figure 2:**
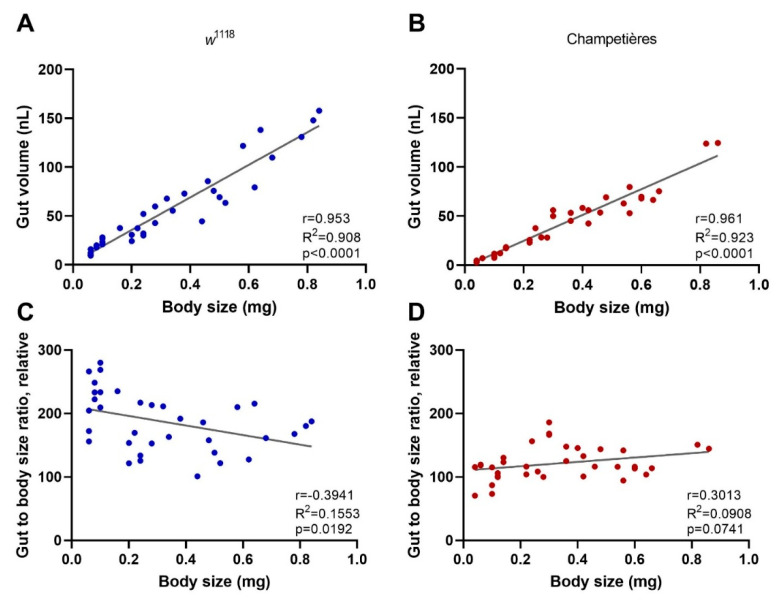
The size of the gut increases in a proportional manner to the total body size: larvae of the *w*^1118^ (blue circles: (**A**,**C**)) and Champetières (red circles: (**B**,**D**)), fly lines of different sizes and instars (L1, L2, and L3) were simultaneously selected and fed with standard solutions of FITC-dextran particles. Groups of 5 similarly sized larvae were weighed, surface cleaned, and macerated, to measure the ingested FITC content and estimate the volume of the gut. The correlation between average weight and average gut volume per group of 5 larvae was estimated using a Pearson correlation test (**A**,**B**), indicating a positive strong correlation between total body and gut size as the larvae grow. Furthermore, the gut-to-body ratio was calculated, and a Pearson test was used to evaluate its correlation to body size (**C**,**D**), indicating no correlation for Champetières, and a weak negative correlation for *w*^1118^ larvae.

One of the key aspects of larval development is constant feeding and growth. We first sought to evaluate whether the larvae ingested proportional amounts of DCV relative to their increasing body size [13]. To do this, we used the total volume of the gut as a proxy for the amount of virus larvae would ingest during a given time, assuming a system at capacity. Larvae of different instars and known body sizes were fed a standard solution of FITC-labelled dextran nanobeads. After 1–2 h the gut was completely filled with the solution, and the solution was constantly being replenished by the constant feeding and excretion. We measured the intensity of the fluorescence signal generated from the FITC within the gut, and estimated the volume by concentration–volume equivalences. We observed a significant positive linear correlation between the larval body mass and the volume of the gut (Pearson r = 0.953 and r = 0.961 for *w*^1118^ and Champetières, respectively; *p* < 0.0001) (Figure 2A,B). Furthermore, we examined whether the ratio between the increasing volume of the gut and the increasing body weight remained constant. This was the case for Champetières larvae (Pearson r = 0.301, *p* = 0.074), whereas it slightly but significantly decreased in *w*^1118^ larvae (Pearson r = −0.394, *p* = 0.019) (Figure 2C,D). The data suggest that the size of the gut maintains near proportionality to the rest of the body as the larva grows, and that larvae would have ingested doses of DCV proportional to their body sizes. This decrease in mortality to a proportional dose indicates that mortality decreases in larger larvae as a result of resistance mechanisms or clearance of infection.

### 3.3. In Adult Flies a Compromised PM Leads to Higher DCV-Induced Mortality 

**Figure 3:**
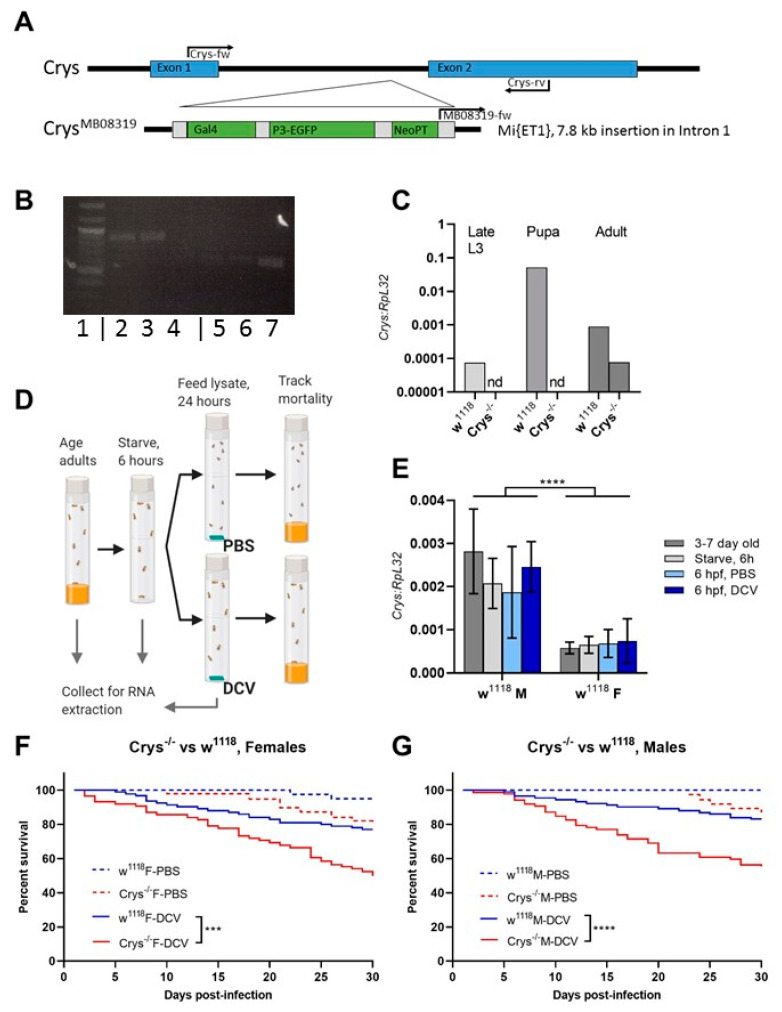
The loss of *Crys* increases the susceptibility to oral viral infection in adult flies: (**A**) the *Crys*^−/−^ line originates from a 7.8 kb Minos-mediated insertion within intron 1 of the *Crys* gene, Mi{ET1} [61,62]. Primers for *Crys* used in this study were designed for differentially amplifying the Mi{ET1} insertion, the wild-type *Crys* gene, and cDNA obtained from processed mRNA. (**B**) The insertion was confirmed by conventional PCR (1: 100bp DNA ladder; 2, 3, 4: wild-type *Crys* gene in *w*^1118^, Champetieres, and *Crys*^−/−^, respectively; 5, 6, 7: *Crys*^MB08319^ in *w*^1118^, Champetières, and *Crys*^−/−^, respectively). (**C**) A decreased or total loss of mRNA expression [24] was confirmed by RT-qPCR in pools of wandering larvae, pupae, or 1-day-old adults of *w*^1118^ and *Crys*^−/−^, and ‘nd’ signifies none-detected. Relative expression of *Crys* to *RpL32* was analysed by ΔΔCt of the estimated efficiency for each primer, each previously calculated from the dilution series of a known, positive sample. (**D**) Adult males and females of 3–7 days-old were briefly starved and orally challenged with PBS- or DCV-lysates mixed with sucrose, and used for RNA extractions and survival bioassays. (**E**) Neither the starvation nor 6 h after having initiated the oral challenge (hours post-feeding, hpf) led to significant differences in the expression of *Crys* mRNA of the wild-type controls [24] when analysed by a two-way ANOVA using Šídák’s multiple comparisons; however, the expression was significantly higher in males than in females at every condition. No *Crys* mRNA was detected in *Crys*^−/−^ adults at any time point of the oral feeding setup (data not shown). (**F**,**G**) After the feeding challenge, we tracked the survival of adults for 30 days. Analysis of survival data indicates that the loss of *Crys* significantly increases the mortality of DCV-fed adults, after the analysis of survival curves using a Cox mixed-effects model (*** *p* < 0.001; **** *p* < 0.0001).

The PM acts as a first physical barrier within the gut, keeping the ingested contents from any physical contact with the epithelial cells, while allowing for the exchange of enzymes and digested products [16,21,26,58]. In *Drosophila*, the major components of the PM are chitin fibres bound as a mesh to long, structural polymers of the Crystallin protein that serve as scaffold [24,59,60]. Loss-of-function *Crystallin* mutant flies (*w*^1118^; Mi{ET1}*Crys*^MB08319^, further referred to as *Crys*^−/−^), have been previously shown to have increased PM permeability, that can lead to bacterial translocation and higher susceptibility to bacterial infections in adult flies [24]. We confirmed the mutation by standard PCR of the Crys gene region, with or without the Mi{ET1} insertion in intron 1 (Figure 3A,B), and by the decreased or loss of expression of *Crys* mRNA in wandering larvae, pupae, and adults of *Crys*^−/−^ flies (Figure 3C). 

To determine whether mortality induced by oral DCV infection of adults is impacted by the loss of Crystallin, *Crys*^−/−^ males and females were orally challenged with DCV and compared to orally challenged *w*^1118^ males and females, as their genetic background counterpart (Figure 3D). Interestingly, we noticed that the ingestion of the virus did not lead to an increase in *Crys* mRNA expression after 6 h of feeding (hpf) with viral particles. This is in contrast to previous reports which indicated a peak of expression in response to pathogenic bacteria 4–8 hpf [24], suggesting the lack of virus- or capsid-specific sensing mechanisms amongst the gut humoral responses. Finally, we observed that the natural expression of *Crys* is significantly higher (*p* < 0.001) in males than in females (Figure 3E), which may be of relevance to further studies that examine the role of the PM over orally acquired infections. Furthermore, we observed a significant increase in mortality in the DCV-fed *Crys*^−/−^ adults, male (Cox-mixed model; coef = −1.25; z = −4.24, *p* = 0.00002) or female (Cox-mixed model; coef = −0.92, z = −3.38, *p* = 0.00072), when compared to their DCV-fed *w*^1118^ counterparts (Figure 3F,G). With these results, we conclude that the loss of *Crys* increases the susceptibility to oral DCV infection in adult flies.

### 3.4. The Loss of Crys Leads to a Compromised PM Phenotype in Older Drosophila Larvae

**Figure 4:**
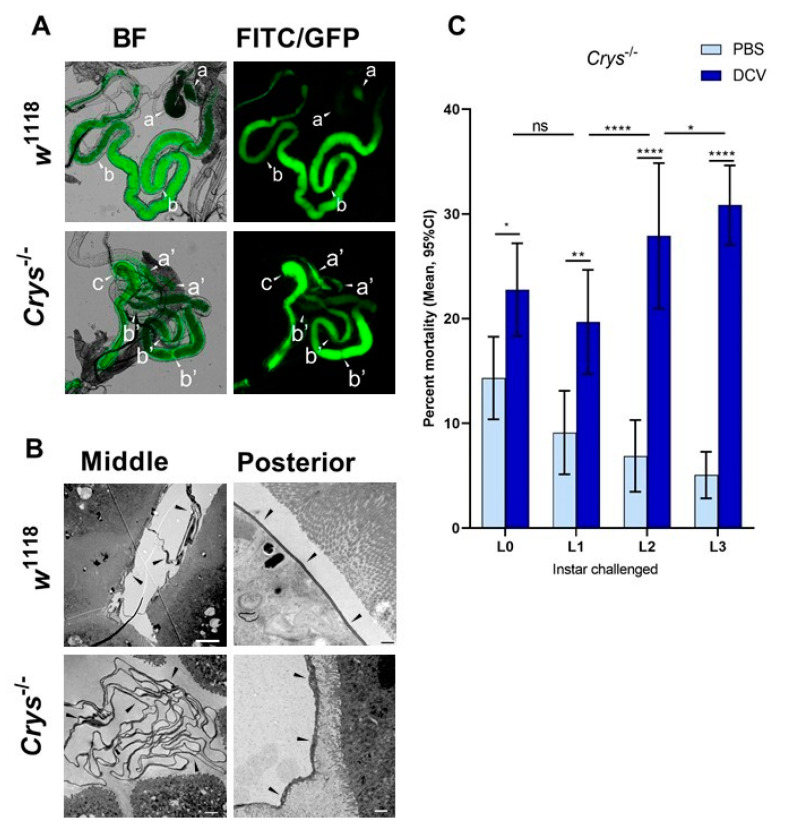
The loss of *Crys* increases the permeability of the larval PM, and increases the mortality of older larvae after oral DCV infection: (**A**) using bright field (BF) and fluorescence microscopy, the crossing of FITC-labelled DCV-sized particles out of the PM and into the gut lumen was observed more commonly in the middle midgut of *Crys*^−/−^ larvae than in their *w*^1118^ counterparts (pointers a, a’). In contrast, the PM of the posterior midgut was permeable to DCV-sized particles in almost every gut analysed (pointers b, b’). The transgenic 3xP3-EGFP marker in *Crys*^−/−^ larvae was observed in the same fluorescence channel used for FITC, and used to confirm the Mi{ET1} cassette insertion. The promoter of this marker is reported to be brain- and eye-specific [64]; however, it also produces a signal in the hindgut (pointer c). (**B**) Using TEM, we evaluated the thickness and structure of the PM (indicated by the pointers) in middle and posterior midguts of L3 larvae of *w*^1118^ and *Crys*^−/−^ larvae. In *Crys*^−/−^, the PM appeared diffuse, irregular, and tended to fold and accumulate in excess in the middle midgut. Bar in *w*^1118^-middle, 5 µm; in *w*^1118^-posterior, 0.5 µm; in both *Crys*^−/−^ images, 1 µm. (**C**) Upon oral challenge following the same protocol as in Figure 1A, we used a two-way ANOVA with Šídák’s test to compare PBS and DCV mortality at each time point. At all time points there was an increased mortality when larvae were fed with DCV lysates. Furthermore, after comparing the effect of PBS or DCV across time points using Tukey’s test, the mortality caused by DCV significantly increased when the oral challenge was done in late larvae (results of statistical analysis are detailed in Table S1; * *p* < 0.05, ** *p* < 0.01, **** *p* < 0.0001).

Having confirmed the increased susceptibility to oral DCV infection in *Crys*^−/−^ adults, and as the previous reports indicated the *Crys*^−/−^ phenotype is characterised by an increased PM permeability [24,59], we tested whether the loss of *Crys* would have a phenotypic impact on the permeability of the larval PM as well. For this, we fed *w*^1118^ or *Crys*^−/−^ L1 and L3 larvae with 500 kDa FITC-labelled dextran beads, with a diameter approximately that of DCV virions [53,54]. After feeding, we dissected the guts and used fluorescence microscopy to quantify the proportion of guts in which the signal from FITC-labelled dextran particles had permeated from the intra-PM space out into the lumen of the three midgut regions: anterior, middle, or posterior (Figure 4A). 

Based on the fluorescence signals detected out of the PM space, the permeability of the PM to DCV-sized particles increases throughout its length, being rare in the anterior midgut, higher in the middle midgut, and occurring in virtually every posterior midgut section we examined (in particular, closest to the midgut–hindgut junction). This pattern was common to either fly line, suggesting that DCV virions might be able to translocate outside of the PM towards the posterior regions of the midgut (Table 2). We found no significant differences in the permeability of the PM to DCV-sized particles in L1 larvae, at any of the three regions measured. In contrast, we found a higher proportion of middle midguts permeable to DCV-sized particles in L3 larvae of *Crys*^−/−^ compared to *w*^1118^ (χ^2^ = 6.76, df = 1, *p* = 0.009). Additionally, we found that the permeability of DCV-sized particles was not significantly different between L1 and L3 larvae of *w*^1118^, whereas it was significantly more common to occur in the middle midguts of L3 than L1 larvae in *Crys*^−/−^ (χ^2^ = 6.81, df = 1, *p* = 0.009). 

Based on these observations, we dissected middle and posterior midguts of L3 *w*^1118^ and *Crys*^−/−^ larvae for visualisation of the PM at either region under TEM. Compared to previous reports that indicated that the loss of *Crys* in adults leads to a thinning of the PM [24], we observed that the PM in *Crys*^−/−^ L3 larvae appears thicker (100–250 nm) than in *w*^1118^ L3 larvae (40–100nm); however, its structure appears irregular and diffuse in *Crys*^−/−^ larvae. Additionally, whereas in the posterior midgut we observed a single layer of PM in either fly line, in the middle midgut the PM folds and accumulates in either line, but to a greater extent in *Crys*^−/−^ larvae (Figure 4B). 

Put together, our PM data suggest that (a) the permeability of the PM remains relatively stable throughout larval development of wild-type larvae; whereas, (b) the permeability of the PM increases in *Crys*^−/−^ larvae as they become older, particularly at the level of the middle midgut, which often appears to be permeable to DCV-sized particles. This increase in permeability in the middle midgut of *Crys*^−/−^ larvae seems to originate from a loose configuration of the PM, which appears thicker as the structural fibres may lack anchor and would occupy a larger volume in the *Crys*^−/−^ line. Additionally, the accumulation of PM folds in *Crys*^−/−^ larvae may slow down the transit of ingested contents, retaining viral particles for longer. In fact, the accumulation of PM in the cardia of adult flies has been associated with intestinal obstruction caused by systemic DCV infection [63].

### 3.5. A Compromised PM Is Associated with Increasing DCV-Induced Mortality of Late Instar Drosophila Larvae

Having confirmed that the *Crys*^−/−^ line has defects in the PM, we repeated the oral DCV challenge experiment at the different larval developmental time points mentioned above. We found that larval mortality was significantly higher than controls after the oral DCV challenge for all time points tested (Šídák’s multiple comparisons for PBS–DCV; *p* = 0.0405 for L0, *p* = 0.0058 for L1, and *p* < 0.0001 for L2 and L3) (Figure 4C, Table S1). Furthermore, whereas in the wild-type lines the difference in mortality between DCV-fed and PBS-fed decreased when challenge occurred in older larvae (Figure 1), in *Crys*^−/−^ larvae this pattern was the opposite: mortality was significantly higher the later in development the DCV challenge occurred (L0 < L1 < L2 < L3; 8% < 11% < 21% < 26%; Table S1). This suggests that the increasing permeability of DCV-sized particles and accumulation of PM in older *Crys*^−/−^ larvae may allow more viral particles to escape out of the PM and reach the gut epithelial cells.

The RNA sequencing data publicly available in GBrowse 2.0 [65] (http://flybase.org/cgi-bin/gbrowse2/dmel/?Search=1;name=FBgn0005664, last accessed 1 January 2021) suggests that mRNA of *Crys* is detected only during the last embryonic stages, before and during pupation, and in adults, but not throughout larval development. Indeed, we did not detect *Crys* mRNA after RNA was extracted from pools of larvae, at any of the four time points tested, for *w*^1118^ or *Crys*^−/−^ with our RT-qPCR protocol (data not shown [48]). Whereas the ratios of chitin, glycoproteins, and proteoglycans in the PM are not well established, it is clear that chitin makes up the overall largest proportion [21,23,32,33]. Chitin-binding proteins, of which *Crys* is the main representative in *Drosophila*, seem to serve mainly for scaffolding and as an anchor to keep the tight mesh structure [24,66]. Furthermore, in *Drosophila* the PM is constantly secreted and this is carried out only by a subset of specialised cells in the cardia [21]. Therefore, RNA extracted from pools of whole larvae may not provide sufficient sensitivity for our RT-qPCR, or in publicly available RNAseq datasets.

### 3.6. The pH of the Gut May Be Able to Inactivate Most DCV Particles Before They Can Escape the PM and Reach the Midgut Epithelium

As mentioned above, based on our observations, the larval midgut in the wild-type *w*^1118^ larvae is most permeable to DCV-sized particles at the posterior region, whereas, in *Crys*^−/−^, it is often permeable to DCV-sized particles in the middle and posterior region. Whether DCV may be able to escape out of the PM becomes important as pH conditions vary throughout the length of the gut, and may be acidic or alkaline enough to affect the stability of the viral capsid [67], degrading the virions as they pass.

**Figure 5:**
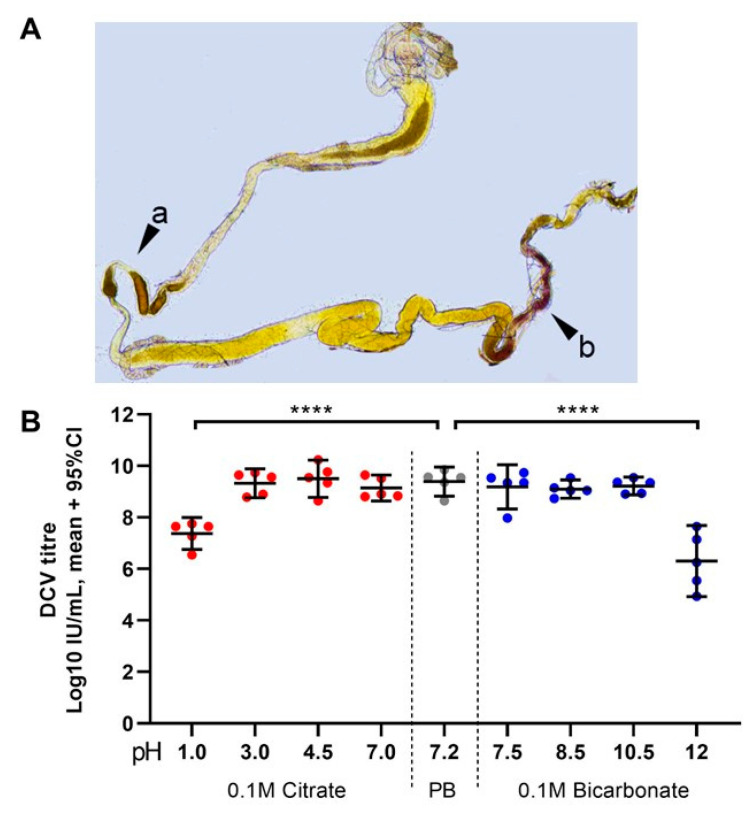
Highly acidic and highly alkaline conditions found in the midgut can affect DCV infectivity: (**A**) upon dissection of L1, L2, and L3 larvae, the *m*-cresol purple ingested shifts to a red/orange hue in the middle midgut indicating a pH between 1.2 and 2.5 (pointer **a**), and to a brown/purple hue towards the end of the posterior midgut, close to the Malpighian tubules, indicating a pH > 9.0 (pointer **b**). These colours fade towards yellow rapidly after dissection, as the gut is neutralised when exposed to the glycerol-PBS. Hence, colours depicted do not accurately reflect those observed in vivo or during dissection; saturation was increased for illustrative purposes. In contrast, the guts of L0 did not display the red or purple colouration of *m*-cresol purple at acid (pH < 2.8) or alkaline (pH > 8.5) conditions, respectively, either before or after dissection (data not shown [48]). (**B**) Aliquots of purified DCV (stock at 2.5 × 10^9^ = 10^9.4^ IU/mL) were incubated under acidic-to-neutral (citrate; left red circles), neutral (PB control; centre grey circles), or neutral-to-alkaline (bicarbonate; right blue circles) buffers for 15 min at room temperature and used to infect *Drosophila* S2 cells. DCV titre was calculated by TCID_50_, and normalised using log-transformation. Each condition was compared to the PB-treated control (in which the purified DCV aliquots were maintained) using Šídák’s multiple comparisons test. Compared to the PB pH 7.2 treatment, highly acidic conditions (Citrate, pH 1.0) decreased the viral titre by 2 orders of magnitude (mean, 10^7.37^ vs. 10^9.39^ IU/mL), whereas highly alkaline conditions (bicarbonate, pH = 12.0) decreased the viral titre by 3 orders of magnitude (mean, 10^6.30^ vs. 10^9.39^ IU/mL) (*p* < 0.0001 for both comparisons). Acidic conditions found within the middle midgut (pH 1–2) may significantly decrease the DCV infectivity, whereas alkaline conditions similar to those found in the posterior midgut (pH 7.5–10.5) are likely not to affect the stability or the remaining infectious viruses.

The pH of the middle midgut of late larvae has been reported to be highly acidic, with values of 2 and below. The posterior region is subdivided in two sections: a neutral zone and an alkaline zone, in which the pH conditions follow a gradient to values above 9 [27] towards the Malpighian tubules and the midgut–hindgut junction. We tested the pH values and gradients at the different regions of the midgut in groups of L0/L1 and L2/L3 larvae of *w*^1118^, Champetières, and *Crys*^−/−^, by feeding them with a pH-sensitive dye, *m*-cresol purple, which appears yellow at neutral pH, and shifts towards orange/red and brown/purple when the pH goes below 2.8 or above 7.5, respectively (Figure 5A). In all fly lines, we observed a highly acidic middle region (pH 1–2), and the pH gradually increased from 7 to more than 9 throughout the length of the posterior midgut in L1, L2, and L3 larvae. Interestingly, in L0 we only observed a yellow colouration throughout the midgut, even before dissection (data not shown [48]), indicating that the pH throughout the midgut was in a range of 3 to 7.5. Furthermore, the acid and alkaline hues of the *m*-cresol purple were visible in our L1 time point, indicating that the acidification and alkalinisation of the midgut probably occurs 6-12 h after the larva has hatched.

Finally, we tested in vitro how DCV infectivity of a purified lab stock (2.5 × 10^9^ IU/mL = 10^9.4^ IU/mL) changes after a 15 min incubation at room temperature in a range of acid and alkaline pH conditions. We found that DCV virions are remarkably stable, maintaining their infectivity in a pH range between 3.0 and 10.5 compared to a DCV control incubated in the same phosphate buffer (PB) pH = 7.2 used for maintaining our lab stocks (Figure 5B). In contrast, highly acidic (pH = 1.0) and highly alkaline conditions (pH = 12.0) led to a mean decrease of 2 and 3 orders of magnitude, respectively, in DCV infectivity compared to the PB control (10^7.3^ IU/mL at pH 1.0; 10^6.4^ IU/mL at pH 12.0; each compared to 10^9.3^ IU/mL of the PB control; Šídák’s multiple comparisons for log-transformed data, *p* < 0.0001 in either case; Figure 5B). These results indicate that the highly acidic conditions of the middle midgut, but not the alkaline conditions of the posterior midgut, play a key role in inactivating the vast majority of ingested viral particles.

## 4. Discussion

Insect-specific viruses are of particular interest as important factors that shape insect communities, and as a means of targeted biocontrol agents [68,69,70,71,72]. Understanding the infectious cycle of these viruses, many of which spread throughout the populations following oral–faecal routes, can help researchers identify potential reservoirs, and the spread and impact over the populations that may become infected. An important aspect of orally acquired insect viruses comes from the constant feeding behaviour of larval stages and developmental resistance to viruses that have been reported across insect families for decades. This resistance can involve increases in lethal dose and/or lethal time between moults, or an almost complete loss of virus-induced mortality upon reaching certain threshold of development [7,12,13,40,73]. However, the mechanisms that underlie these patterns of resistance are not fully understood.

We aimed to study which mechanisms contribute to the increase in resistance seen in larvae of *D. melanogaster* orally challenged with its most pathogenic virus, the dicistrovirus DCV. Our results confirm earlier observations of a decrease in the mortality of larvae when infection occurs later in development [40], similar to other insect–virus models [7,8,12,73]. As the increase in larval size can have effects over the necessary viral dose for infection and lethality, we estimated the gut-to-body ratio, and found that this remains relatively stable as the larva grows, indicating that in our experiments larvae were fed equivalent doses for their size. This suggests that either (a) similar proportions of larvae become infected at each time point, but older instars do not die from, or may clear the infection; or (b) ageing larvae develop mechanisms that prevent them from becoming infected.

Like many other insects, the midgut of *Drosophila* is protected from ingested pathogens by a type II PM, constitutively secreted by the cardia [21]. In adult *Drosophila*, this complex structure of chitin and glycoproteins, from which Crystallin is among the major structural components, has been previously demonstrated to be necessary for effective antibacterial protection [24,59]. We obtained the same Crystallin-deficient line used in the previous study (referred to as *dcy*^1^ in Kuraishi et al. [24]), based on the evidence presented for the increased permeability and decreased thickness of the PM, and tested it in the context of viral infection. Similar to bacterial infections, we observed that adult flies with a weakened PM have increased mortality upon oral DCV infection, suggesting that this line could be an appropriate model for our study. The increase in permeability that occurs later in development within this fly line may be directly associated with the increasing mortality following the oral DCV challenge to older larvae, as the larval PM would protect the gut epithelium from infection by physical containment of the ingested viral particles. Interestingly, we observed that whereas the PM in *Crys*^−/−^ larvae is more permeable to DCV-sized particles than their wild-type controls, it appears thicker, but more diffuse and irregular. Polymers of Crys mainly serve as support for the chitin fibres of the PM, by providing anchor for fibre bundles, and overall structural support and rigidity [59]. Even if the basal expression of *Crys* in larvae appears to be below levels of detection using conventional RT-qPCR techniques, the differential phenotypes we observed point to the Crys protein as a key regulator of the PM, and a composition that is different between larvae and adults.

Apart from the containment of ingested materials the PM provides, the acid and alkaline conditions within the midgut also serve as strong barriers against pathogens [27,74]. The acid conditions of the middle midgut, where we and others have noted pH values below 2 [27,75], would be capable of inactivating the majority of the ingested DCV virions, according to our in vitro results. Interestingly, we observed strongly acidic and alkaline conditions within the midgut starting from our L1 time point (~12–18 h old, and older), but not in L0 (<6 h old). This suggests that the gut of the larva soon after hatching may not inactivate the ingested DCV virions as effectively as the gut of older larvae and could explain part of the high mortality in the earlier time points. In fact, the PM in the posterior midgut was permeable enough for full virions to escape in ~95% of all samples tested in our FITC-dextran particles assay, so chemical inactivation may be key in the antiviral defence of the gut.

Previous physical–chemical analyses of the Triatoma virus [67], another Dicistrovirus, indicate that a pH between 8 and 9 is enough to destabilise the capsid subunits, and release the viral genome. Interestingly, we found that DCV virions were able to remain infectious in a pH up to 10.5, suggesting the posterior midgut, where the pH can reach values of 10–11 [27,75], may not effectively inactivate the virions that would have passed intact through the middle midgut. It would be interesting to consider if, similar to the high alkalinity required for degrading the occlusion bodies of baculoviruses [76], high alkalinity may be necessary for destabilising or inducing conformational changes to the capsid of dicistroviruses, facilitating the binding to the cell membrane or the release of its genome into the gut epithelial cells, as it has been discussed by physical–chemical studies [67]. It is also interesting to consider whether the alkaline conditions may favour or hinder the clathrin-mediated endocytosis processes, necessary for infection by DCV [77]. However, these processes in the infectious cycle of DCV and other dicistroviruses remain largely unknown. Additionally, little information is available regarding the pH conditions of the larval gut in *Drosophila*, beyond that of L3 larvae. Determining when the acidification and alkalinisation of the larval midgut occurs, and which mechanisms drive them, might be of relevance for further research on the biology of the gut defences.

Put together, our data suggest that, upon ingestion of food contaminated with DCV, the PM serves as a first physical barrier, preventing ingested virions from reaching the midgut epithelium for approximately half to two-thirds of its total length. Furthermore, we hypothesised that a large proportion of infectious DCV particles would be inactivated, whilst being contained within the PM, by the strong acid conditions of the middle midgut. Indeed, as the middle midgut becomes more permeable in older *Crys*^−/−^ larvae, we observed an increase in mortality, potentially coming from still-stable DCV particles accumulating in the PM folds, escaping earlier, and rapidly establishing infection in this region. Finally, upon reaching the posterior midgut, in which the neutral-to-alkaline gradient occurs, along with the PM permeability naturally increasing in this region, any remaining DCV particle that is still infectious, may at last reach the epithelium and establish an infection. It is noteworthy to consider the digestive enzymes to which DCV virions are also exposed while transiting the gut, and cell- and tissue-specific antiviral defences and repair mechanisms can occur as a response to infection. Such interplay of defences may explain the low rates of infection and mortality seen overall in our experimental settings, and in other research focused on the oral route of viral infection in *Drosophila* larvae and adults [41,42,78,79]. Whereas the mechanisms proposed here may apply to DCV and other small, non-enveloped RNA viruses, it is important to consider how our model may extrapolate to other insect–virus pairs. Indeed, the alkalinisation in the posterior midgut is necessary in the infectious cycle of baculoviruses in lepidopterans for releasing the virions from the occlusion bodies and initiate infection [80], and bacterial-, parasite-, and baculovirus-specific proteases and chitinases that degrade the PM [31,81,82] act as virulence factors that are not present in picornaviruses. Further research using lepidopteran models, or the recently described Drosophila nudivirus within its *Drosophila* host [83], may clarify which gut-specific mechanisms, and in what contexts, can be anti- or pro-viral, and point to how they may be best utilised in the fields of agriculture, conservation, and medicine.

## Figures and Tables

**Table 1:** Primers used in this study.

	Sequence	Product Length
**Conventional PCR**		
12SA1	5′-AAACTAGGATTAGATACCCTATTAT-3′	400 bp
12SB1	5′-AAGAGCGACGGGCGATGTGT-3′	
Wsp-81F	5′-TGGTCCAATAAGTGATGAAGAAAC-3′	610 bp
Wsp-691R	5′-AAAAATTAAACGCTACTCCA-3′	
Crys-Fw	5′-CATCGGCAGCAAACGGAAAA-3′	897 bp ^a^
Crys-MB08319-Fw	5′-ATGAAAGGTTGGGCTTCGGA-3′	642 bp ^b^
Crys-Rv	5′-GACGCAGGTATGCCGAATTG-3′	
**RT-qPCR**		
Rpl32-Fw	5′-GACGCTTCAAGGGACAGTATCTG-3′	141 bp
RpL32-Rv	5′-AAACGCGGTTCTGCATGAG-3′	
Crys-Fw	5′-CATCGGCAGCAAACGGAAAA-3′	150 bp ^c^
Crys-Rv	5′-GACGCAGGTATGCCGAATTG-3′	

^a^ Obtained using Crys-Fw/Crys-Rv and genomic DNA. ^b^ Obtained with Crys-MB08319-Fw/Crys-Rv on genomic DNA, *Crys*^−/−^-specific. ^c^ Obtained using Crys-Fw/Crys-Rv and cDNA synthesized from mature mRNA. Amplification using the Crys-Fw/Crys-Rv primer pair in *Crys*^−/−^ flies may generate fragments 7.8 kb longer than those obtained from *w*^1118^ flies, when long-product PCR reactions are carried out.

**Table 2:** The middle midguts of old *Crys*^−/−^ larvae is more often permeable to DCV-sized particles.

FITC Outside PM		Anterior Midgut	Middle Midgut	Posterior Midgut
**L1**	***w*** **^1118^**	4.9% (2/41)	19.5% (8/41)	92.7% (38/41)
	***Crys*** **^−/−^**	6.1% (2/33)	30.3% (10/33)	93.9% (31/33)
**L3**	***w*** **^1118^**	7.9% (3/38)	31.5% (12/38)	94.7% (36/38)
	***Crys*** **^−/−^**	16.3% (7/43)	60.5% (26/43)	97.7% (42/43)
		**χ^2^**	**z**	***p***
**L1 vs. L3, *w*^1118^**				
Anterior		0.303	0.550	0.582
Middle		1.519	1.232	0.218
Posterior		0.140	0.374	0.708
**L1 vs. L3, *Crys*^−/−^**				
Anterior		1.867	1.367	0.172
Middle		**6.813**	**2.610**	0.009 *
Posterior		0.687	0.829	0.407
***Crys*** **^−/−^** ** vs. *w*^1118^, L1**				
Anterior		0.050	0.223	0.823
Middle		1.157	1.075	0.282
Posterior		0.046	0.214	0.831
***Crys*** **^−/−^** ** vs. *w*^1118^, L3**				
Anterior		1.310	1.145	0.252
Middle		**6.759**	**2.600**	0.009*
Posterior		0.488	0.698	0.485

The number of midgut regions of L1 and L3 larvae of *w*^1118^ and *Crys*^−/−^ flies that were permeable to DCV-sized FITC-dextran particles were compared between instars or between lines using χ^2^ tests.

## Supplementary Materials

The following are available online at https://www.mdpi.com/article/10.3390/v13050894/s1, Table S1: statistical analyses for mortality of *w*^1118^, Champetières, and *Crys*^−/−^ larvae upon oral DCV challenge.
