# Supplementary material for: Physical and Chemical Barriers in the Larval Midgut Confer Developmental Resistance to Virus Infection in Drosophila
Source: Viruses. 2021 May 12;13(5):894. doi: 10.3390/v13050894 (PMC8151258; doi:10.3390/v13050894)
Supplement: Supplementary file 1 [file viruses-13-00894-s001.zip › viruses-1188178-supplementary.pdf]

Table S1. Statistical analyses for mortality of w^{1118}, Champetières and $Crys^{-/-}$ larvae upon oral DCV challenge

w^{1118}

Ordinary Two-way ANOVA, Alpha 0.05

**Source of Variation**	**% of total variation**	**P value**
Interaction	8.736	0.0001 ***
Instar	20.59	<0.0001 ****
Infection	37.78	<0.0001 ****

**Šídák's multiple comparisons test**	**Predicted (LS) mean 1**	**Predicted (LS) mean 2**	**Predicted (LS) mean diff.**	**95.00% CI of diff.**	**Adjusted P Value**
PBS - DCV					
L0	7.842	33.05	-25.21	-33.59 to -16.84	<0.0001 ****
L1	8.582	32.45	-23.87	-32.24 to -15.49	<0.0001 ****
L2	5.668	17.26	-11.59	-19.97 to -3.217	0.0029 **
L3	2.892	9.151	-6.258	-14.63 to 2.117	0.2200

**Tukey's multiple comparisons test**	**Predicted (LS) mean 1**	**Predicted (LS) mean 2**	**Predicted (LS) mean diff.**	**95.00% CI of diff.**	**Adjusted P Value**
PBS					
L0 vs. L1	7.842	8.582	-0.7402	-10.19 to 8.705	0.9969
L0 vs. L2	7.842	5.668	2.174	-7.271 to 11.62	0.9301
L0 vs. L3	7.842	2.892	4.949	-4.496 to 14.39	0.5170
L1 vs. L2	8.582	5.668	2.914	-6.531 to 12.36	0.8489
L1 vs. L3	8.582	2.892	5.689	-3.756 to 15.13	0.3940
L2 vs. L3	5.668	2.892	2.775	-6.669 to 12.22	0.8664
DCV					
L0 vs. L1	33.05	32.45	0.6017	-7.110 to 8.313	0.9969
L0 vs. L2	33.05	17.26	15.79	8.080 to 23.50	<0.0001 ****
L0 vs. L3	33.05	9.151	23.9	16.19 to 31.61	<0.0001 ****
L1 vs. L2	32.45	17.26	15.19	7.479 to 22.90	<0.0001 ****
L1 vs. L3	32.45	9.151	23.3	15.59 to 31.01	<0.0001 ****
L2 vs. L3	17.26	9.151	8.109	0.3977 to 15.82	0.0355 *

Champetières

Ordinary Two-way ANOVA, Alpha 0.05

Source of Variation	% of total variation	P value
Interaction	11.86	<0.0001 ****
Instar	19.02	<0.0001 ****
Infection	41.13	<0.0001 ****

Šídák's multiple comparisons test	Predicted (LS) mean 1	Predicted (LS) mean 2	Predicted (LS) mean diff.	95.00% CI of diff.	Adjusted P Value
PBS - DCV					
L0	8.537	36.08	-28.25	-34.33 to -22.17	<0.0001 ****
L1	6.999	26.38	-19.38	-25.46 to -13.30	<0.0001 ****
L2	9.336	21.81	-12.48	-18.55 to -6.396	<0.0001 ****
L3	4.892	9.553	-4.661	-10.74 to 1.418	0.2008

Tukey's multiple comparisons test	Predicted (LS) mean 1	Predicted (LS) mean 2	Predicted (LS) mean diff.	95.00% CI of diff.	Adjusted P Value
PBS					
L0 vs. L1	8.537	6.999	1.538	-5.320 to 8.397	0.9358
L0 vs. L2	8.537	9.336	-0.7987	-7.658 to 6.060	0.9901
L0 vs. L3	8.537	4.892	3.645	-3.214 to 10.50	0.5085
L1 vs. L2	6.999	9.336	-2.337	-9.196 to 4.522	0.8092
L1 vs. L3	6.999	4.892	2.106	-4.753 to 8.965	0.8526
L2 vs. L3	9.336	4.892	4.443	-2.416 to 11.30	0.3321
DCV					
L0 vs. L1	36.79	26.38	10.41	4.808 to 16.01	<0.0001 ****
L0 vs. L2	36.79	21.81	14.98	9.379 to 20.58	<0.0001 ****
L0 vs. L3	36.79	9.553	27.24	21.64 to 32.84	<0.0001 ****
L1 vs. L2	26.38	21.81	4.571	-1.029 to 10.17	0.1495
L1 vs. L3	26.38	9.553	16.83	11.23 to 22.43	<0.0001 ****
L2 vs. L3	21.81	9.553	12.26	6.658 to 17.86	<0.0001 ****

Crys$^{-/-}$

Ordinary Two-way ANOVA, Alpha 0.05

**Source of Variation**	**% of total variation**	**P value**
Interaction	8.96	0.0004 ***
Instar	1.766	0.2764
Infection	46.95	<0.0001 ****

**Šídák's multiple comparisons test**	**Predicted (LS) mean 1**	**Predicted (LS) mean 2**	**Predicted (LS) mean diff.**	**95.00% CI of diff.**	**Adjusted P Value**
PBS - DCV					
L0	14.33	22.77	-8.442	-16.63 to -0.2552	0.0405 *
L1	9.112	19.69	-10.58	-18.76 to -2.390	0.0058 **
L2	6.878	27.91	-21.03	-29.22 to -12.85	<0.0001 ****
L3	5.055	30.84	-25.79	-33.98 to -17.60	<0.0001 ****

**Tukey's multiple comparisons test**	**Predicted (LS) mean 1**	**Predicted (LS) mean 2**	**Predicted (LS) mean diff.**	**95.00% CI of diff.**	**Adjusted P Value**
PBS					
L0 vs. L1	14.33	9.112	5.216	-4.021 to 14.45	0.4551
L0 vs. L2	14.33	6.878	7.45	-1.787 to 16.69	0.1575
L0 vs. L3	14.33	5.055	9.273	0.03646 to 18.51	0.0487 *
L1 vs. L2	9.112	6.878	2.234	-7.003 to 11.47	0.9212
L1 vs. L3	9.112	5.055	4.057	-5.179 to 13.29	0.6600
L2 vs. L3	6.878	5.055	1.823	-7.413 to 11.06	0.9549
DCV					
L0 vs. L1	22.77	19.69	3.081	-4.460 to 10.62	0.7091
L0 vs. L2	22.77	27.91	-5.14	-12.68 to 2.402	0.2879
L0 vs. L3	22.77	30.84	-8.073	-15.61 to -0.5313	0.0310 *
L1 vs. L2	19.69	27.91	-8.221	-15.76 to -0.6797	0.0270 *
L1 vs. L3	19.69	30.84	-11.15	-18.70 to -3.613	0.0011 **
L2 vs. L3	27.91	30.84	-2.933	-10.47 to 4.609	0.7396

We used a Two-way ANOVA to analyse the proportion of morality after PBS- or DCV-feeding larvae at four time points (L0, L1, L2, L3, see Methods). Multiple comparisons were made with Šídák’s test between PBS and DCV survival, at each of the four time points defined in our infection protocol (comparisons within); and with Tukey’s test to compare

the survival of instars when challenged with either PBS or DCV lysates (comparisons between).
